# Satellite Flood Inundation Assessment and Forecast Using SMAP and Landsat

**DOI:** 10.1109/JSTARS.2021.3092340

**Published:** 2021-06-25

**Authors:** Jinyang Du, John S. Kimball, Justin Sheffield, Ming Pan, Colby K. Fisher, Hylke E. Beck, Eric F. Wood

**Affiliations:** Numerical Terradynamic Simulation Group, University of Montana, Missoula, MT 59801 USA; School of Geography and Environmental Sciences, University of Southampton, Southampton SO17 1BJ, U.K.; Civil and Environmental Engineering, Princeton University, Princeton, NJ 08542 USA, and also with the Center for Western Weather and Water Extremes, Scripps Institution of Oceanography, University of California San Diego, La Jolla, CA 92093 USA; Princeton Climate Analytics, Princeton, NJ 08542 USA; Civil and Environmental Engineering, Princeton University, Princeton, NJ 08542 USA, and also with the European Commission, Joint Research Centre (JRC), 21027 Ispra, Italy; Civil and Environmental Engineering, Princeton University, Princeton, NJ 08542 USA

**Keywords:** Flood, Global Forecast System (GFS), Google Earth Engine (GEE), Landsat, Soil Moisture Active Passive (SMAP)

## Abstract

The capability and synergistic use of multisource satellite observations for flood monitoring and forecasts is crucial for improving disaster preparedness and mitigation. Here, surface fractional water cover (FW) retrievals derived from Soil Moisture Active Passive (SMAP) L-band (1.4 GHz) brightness temperatures were used for flood assessment over southeast Africa during the Cyclone Idai event. We then focused on five subcatchments of the Pungwe basin and developed a machine learning based approach with the support of Google Earth Engine for daily (24-h) forecasting of FW and 30-m inundation downscaling and mapping. The Classification and Regression Trees model was selected and trained using retrievals derived from SMAP and Landsat coupled with rainfall forecasts from the NOAA Global Forecast System. Independent validation showed that FW predictions over randomly selected dates are highly correlated (*R* = 0.87) with the Landsat observations. The forecast results captured the flood temporal dynamics from the Idai event; and the associated 30-m downscaling results showed inundation spatial patterns consistent with independent satellite synthetic aperture radar observations. The data-driven approach provides new capacity for flood monitoring and forecasts leveraging synergistic satellite observations and big data analysis, which is particularly valuable for data sparse regions.

## I. Introduction

Extreme rainfall-driven flooding is one of the most widespread and costly natural disasters [1] and is expected to become more frequent with global warming [2]. As one of the deadliest and most devastating storms on record in the southern hemisphere, tropical cyclone Idai brought extreme rainfall to southeast Africa in March 2019, affecting about 3 million people, damaging more than 200 000 houses and resulting in more than 1000 deaths and total damages exceeding $2 Billion [3]. Timely assessment and early warning systems are essential to disaster preparedness and rapid responses. Advances in remote sensing and big data techniques provide new opportunities for building efficient and effective all-weather and multiscale flood assessment and forecast capabilities.

Satellite optical-infrared (IR) and microwave remote sensing observations are suitable for delineating flood inundation extent over large areas due to the unique surface reflectance and microwave signatures of standing water [4]. Satellite optical-IR sensors such as PlanetScope multispectral cameras, Landsat, and MODIS enable accurate detection of open water at submeter to 1000-m spatial resolutions and global coverage at daily to 16-day cycles [5]–[7]. However, cloud cover and suboptimal solar illumination can severely reduce the number of valid measurements from optical-IR remote sensing, resulting in major data loss during rainfall-driven flood events [8]. Despite the drawbacks likely limiting near-real-time flood monitoring, long-term water inundation records composited from clear-sky optical-IR observations are valuable in quantifying historical water inundation dynamics and flood feasibility [9], [10].

Microwave remote sensing is another powerful tool for flood monitoring due to the strong microwave sensitivity to surface water, and relative insensitivity to solar illumination, atmosphere, and cloud cover constraints [11]. In addition, microwave signals are more capable of detecting water features under vegetation relative to optical-IR observations, although the degree of vegetation contamination and signal loss is proportional to channel frequency, with greater vegetation transparency and surface water sensitivity at lower microwave frequencies [11], [12]. Active microwave remote sensing allows for flood mapping under all-weather conditions at resolutions on the order of meters to a few kilometers [13]–[16] but with infrequent monitoring provided from existing satellite synthetic aperture radar (SAR) based observations (e.g., approximately six-day global coverage for Sentinel-1 constellations) or limited spatial coverage from global navigation satellite (GNSS) based techniques (e.g., areas between 38° N and 38° S latitude for Cyclone GNSS constellation) [17].

Passive microwave radiometry has also been used for flood mapping and provides capabilities for global monitoring with high temporal frequency (~1–3 days) but at coarse (5–25 km) spatial scales [18]–[21]. For example, the National Aeronautics and Space Administration (NASA) Soil Moisture Active Passive (SMAP) and European Space Agency (ESA) Soil Moisture and Ocean Salinity (SMOS) missions provide low frequency (L-band) microwave emission observations with enhanced sensitivity to water signals underlying vegetation [22], [23], though potential applications requiring finer landscape level assessments of surface water dynamics are limited by the coarse (~40 km) SMAP footprint [21].

Due to the complementary nature of different remote sensing techniques, data fusion approaches combining multisensor observations show promise for enhanced flood mapping in terms of accuracy, temporal coverage, and spatial resolution [24], [25]. The emergence of cloud-based geospatial processing platforms such as Google Earth Engine (GEE) provides an efficient means for rapid access and combined analysis of multisource data [26]. The capability of accurate flood mapping within minutes was achieved by analyzing hundreds of Sentinel-1 SAR and Landsat images archived on the GEE [27]–[29]. In addition to exploiting a growing number of observations from current satellite sensors through big data techniques, planned next generation satellite missions including the NASA-ISRO SAR and NASA-CNES SWOT radar altimetry missions will enable further enhancement in global water cycle and flood assessment leveraging satellite river gauging and high spatial–temporal resolution observations [30], [31].

While timely satellite assessment is crucial to disaster emergency response at the time of flooding, effective flood inundation forecasts are indispensable for early warning systems, disaster preparedness, and management. Traditional flood forecast systems exploit flood-related hydrologic processes simulated by physical models, which rely on quantified descriptions of catchment and river physical characteristics, and are driven by rainfall outputs from a numerical weather prediction (NWP) model [32]–[34]. For example, a flood forecasting system utilizing graphics processing unit computation showed potential in predicting water level and flood extent with 34 h of lead time for a selected catchment [35]. Considering the highly nonlinear correspondence between rainfall and flood inundation, and the lack of accurate descriptions of hydrologic parameters at sub-kilometer levels, data-driven approaches represent an alternative to physically based forecast systems by leveraging the flexibility of machine-learning methods in linking rainfall inputs and inundation outputs [36]–[38]. Despite recent advances in empirical data-driven flood forecasts, direct flood observations (e.g., inundation extent) from satellites have not been comprehensively utilized in current forecast systems. The flood inundation pattern inherent in long-term satellite observations has also not been fully utilized to inform regional flood forecasts. The capabilities of efficient and fine-scale (e.g., 30-m) flood inundation forecasts targeting individual houses or small neighborhoods are still lacking, especially for data sparse regions where effective preflood disaster preparedness and risk mitigation are greatly needed.

Here, we used global NASA SMAP surface fractional water cover (FW) observations [21] for monitoring flood inundation during the cyclone Idai event over southeast Africa. We developed a machine-learning scheme for obtaining finer (30-m) resolution flood forecasts by fusing synergistic information from satellite observations and NWP outputs. Detailed descriptions of the methods, results, and discussion from this article are presented in Sections II, III, and IV, respectively.

## II. Methods

### A. Study Region

**Fig. 1.**
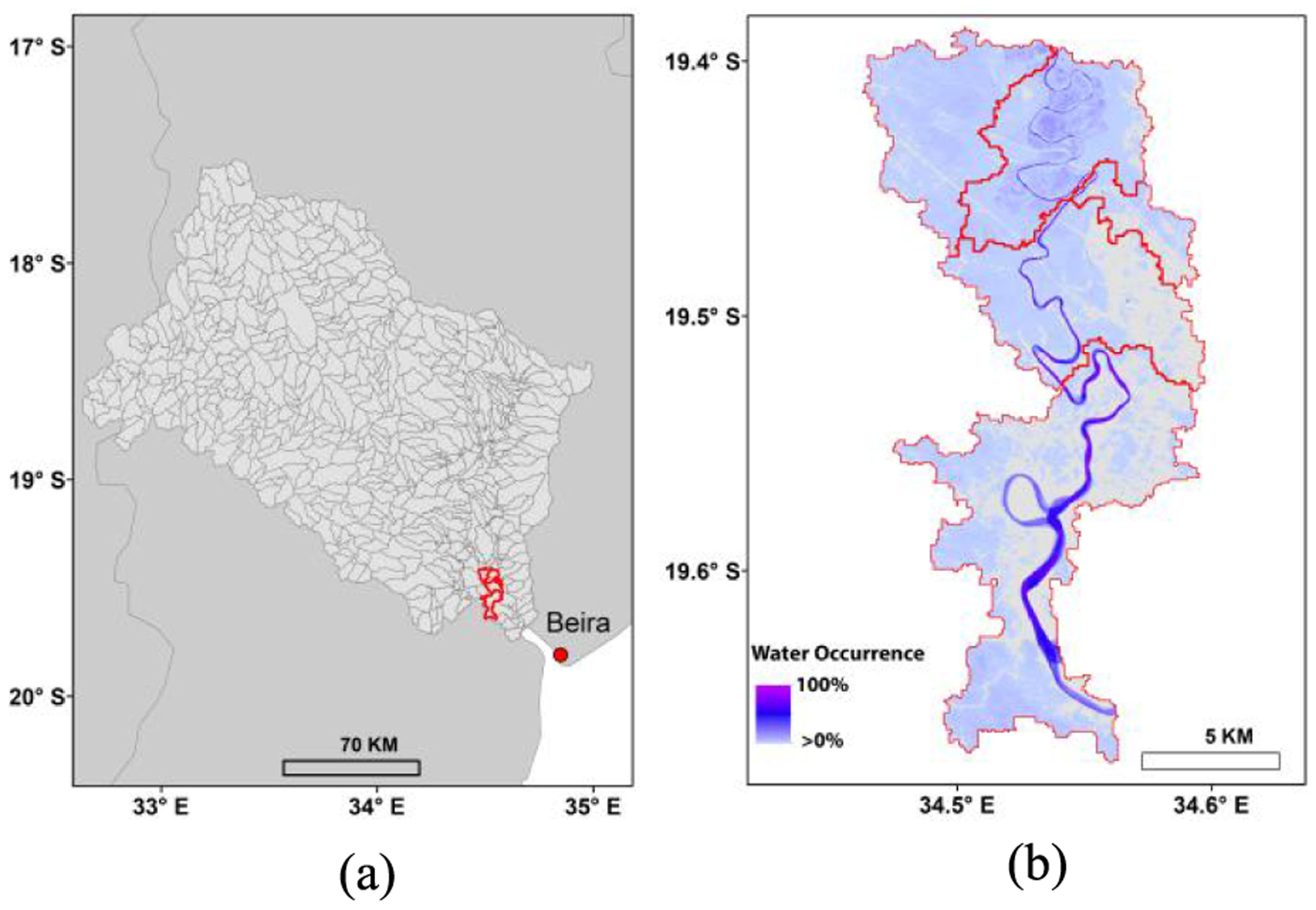
(a) Five unit catchments (delineated in red) within the Pungwe basin and (b) water occurrence from 2000 to 2019 over the catchments derived from the USGS Landsat water mask.

Our study involves regional flood mapping over southeast Africa (latitude: −5° to −35°; longitude: 18° to 50°), along with finer (30-m) scale flood inundation forecast assessments over five unit catchments (~163 km^2^) within the lower Pungwe River basin [Fig. 1(a)]. The basin covers ~31 000 km^2^ extending from Zimbabwe’s eastern highlands to the Sofala province lowlands in Mozambique; the region experiences seasonal wet and dry cycles with recurring drought and flood events [39]. Cyclone Idai made landfall near Beira, the Sofala provincial capital, on the night of March 14 to March 15, 2019, as a category 2 storm [40]. As the storm moved slowly inland, it brought extreme rainfall that led to devastating flooding in Mozambique and triggered major flooding over the larger southeast African region [40]. Regional flooding was exacerbated by persistent rainfall and wet conditions in the weeks prior to the Idai event. The flood inundation distributions over southeast Africa were mapped using SMAP FW data in our study. For evaluating the potential utility of satellite-based flood forecasts, we focused on the five unit catchments of the Pungwe basin [Fig. 1(b)] within the Sofala province, where severe flooding occurred during the Idai event [40].

### B. Datasets

Five dynamic datasets were used in this study including the SMAP FW record [21], the NASA-United States Department of Agriculture (USDA) SMAP global soil moisture dataset [41], [42], United States Geological Survey (USGS) Landsat water mask data [43], National Oceanic and Atmospheric Administration (NOAA) National Centers for Environmental Prediction (NCEP) Global Forecast System (GFS) 384-Hour Predicted Atmosphere Data [44], and NASA Advanced Rapid Imaging and Analysis (ARIA) flood inundation products [45], [46]. One static dataset, depicting unit catchment boundaries from the Multi-Error-Removed-Improved-Terrain (MERIT) Basins dataset [47], was also used for the study. The SMAP FW and MERIT boundary data were uploaded to GEE in this study for performing the multisource analysis with the other datasets, which are accommodated and regularly updated on GEE. Except for the static MERIT boundary data, all other datasets used in this study were temporally dynamic.

The FW data derived using SMAP brightness temperature (Tb) observations represent the areal proportion of standing water within the sensor footprint (~40 km resolution) [21]. The SMAP mission was successfully launched in January 2015 and provides desirable characteristics for FW monitoring, including L-band (1.4 GHz) microwave sensitivity to surface water and reduced sensitivity to atmosphere contamination and overlying vegetation cover relative to optical-IR and higher-frequency microwave satellite observations, consistent sensor view geometry, well-calibrated Tb retrievals, and advanced detection and mitigation of radio frequency interference [22]. The SMAP FW annual averages are highly correlated (*R* = 0.85) with alternative global water maps derived from MODIS (MOD44W) observations, while capturing both flash flooding and seasonal inundation variations from 1–3 day global coverage [21]. The SMAP ascending orbit FW data from March 11 to 19, 2019 were used directly for regional mapping of the Idai flood event; and the multiyear record (July 2015 to March 2019) was used along with Landsat and GFS records for the flood forecasts by accounting for the surface water conditions prior to the forecast dates. We used FW retrievals from SMAP ascending orbits due to their higher accuracy relative to the alternative estimates derived from descending orbit observations [21].

The NASA-USDA SMAP global soil moisture dataset is generated by assimilating SMAP surface soil moisture into the modified two-layer Palmer model for providing both surface and subsurface soil moisture over the globe at 0.25° × 0.25° spatial resolution [41]. The water-holding capacity of saturated soil in the surface layer is assumed to be 25.4 mm [41]. The resulting soil moisture product showed improved correlation with *in situ* measurements relative to model outputs derived without assimilating SMAP products [42]. Here, the surface soil moisture data were used to depict background soil wetness conditions prior to the target prediction date for the flood forecasts.

The 30-m Landsat water mask data integrated in the USGS Landsat-7/8 surface reflectance products [48] were used to calculate the FW of the selected unit catchments and served as the target variable in the flood forecast model. The Landsat water mask data were originally derived using the Fmask algorithm [48], which has been widely used with optical-IR imagery for distinguishing land, water, cloud, and cloud shadow, with a documented 2% omission error and 14% commission error [43]. For this study, only Landsat observations with cloud coverage less than 20% were selected to calculate the reference FW values for training and validating the forecast model. In addition, the Landsat data from 2000 to 2019 were used to generate water occurrence data, which represents overall flood feasibility for the past two decades. For example, floods have frequently occurred in the selected unit catchments as evidenced by the widespread distribution of areas with high water occurrence [e.g., >30% highlighted in light blue to purple; Fig. 1(b)].

The GFS is a three-dimensional weather forecast model operationally running at NOAA-NCEP [49] and archived on GEE for the record since July 2015. The GFS couples a variety of models accounting for atmosphere, ocean, land, and sea ice processes, and provides up to 384-h forecasts, with 3-h forecast intervals for selected model outputs as gridded forecast variables [49]–[51]. The GFS precipitation forecasts have been coupled with hydrological models to improve runoff predictions [50] and understanding of hydrological processes [52]. The GFS forecasts of cumulative surface precipitation at 0.25° spatial resolution served as predictors for deriving the flood inundation forecast. We also used GFS precipitation outputs to describe background rainfall conditions prior to the forecast date.

The catchment boundary delineations were derived from MERIT hydrography data [47], which account for topographic effects using a 3-arcsec (~90 m) resolution DEM [53]. The MERIT Basins dataset provides enhanced delineation of unit catchments over the globe, including approximately 2.94 million vectorized river flowlines and unit catchments [47]; these data provided the required hydrography for the river routing and hydrological simulations from this study.

Flood maps independently derived by the NASA Jet Propulsion Laboratory ARIA project [47] using space-borne SAR observations were used for assessing the inundation forecast results from this study. ARIA flood proxy maps for March 19 and March 23, 2019 over Mozambique were produced using imagery acquired by Sentinel-1 SAR and the Phased Array type L-band SAR (PALSAR) onboard the Advanced Land Observing Satellite 2 (ALOS-2), respectively. The ARIA maps delineated areas likely flooded due to Cyclone Idai at a spatial resolution of 30 m for Sentinel-1 and 25 m for ALOS-2 results. The ALOS-2 flood maps were resampled to 30-m resolution for comparing against the Sentinel-1 and model forecast flood results from this study. The processed images were compared to each other for cross-validation, while larger differences and uncertainties in the satellite derived flood maps are expected over urban and vegetated areas [46].

### C. Regional Flood Mapping Using SMAP

Regional flood mapping was performed by analyzing the SMAP derived FW dynamics. For deriving SMAP FW data, an ancillary lookup table (LUT) was first established to provide reference L-band microwave emissivities for land and water end-members, excluding ocean areas, under a range of land surface conditions defined by an existing Advanced Microwave Scanning Radiometer (AMSR) global land parameter data record [21], [54]. Land and water endmembers for the LUT were identified as grid cells fully (100%) land and fully water covered using an ancillary global land cover map and the AMSR land parameter record. Based on the ancillary LUT and using SMAP daily ascending orbit *T*_*b*_ (L1CTB) retrievals as primary inputs, daily FW retrievals were derived over the global domain using a difference ratio (DR) of SMAP emissivities [21]

(1) $$\text{FW}=\frac{\left(e_{hl}^{\text{ref}}-e_{h}^{\text{obs}}\right)}{\left(e_{hl}^{\text{ref}}-e_{hw}^{\text{ref}}\right)}\approx \frac{\left(T_{bhl}^{\text{ref}}-T_{bh}^{\text{obs}}\right)}{\left(T_{bhl}^{\text{ref}}-T_{bhw}^{\text{ref}}\right)}$$

where *h* denotes *H-polarization*, *l* is for pure land, *w* is for pure water, ref is the reference emissivity (or *T*_*b*_) under the LUT defined land surface condition, and obs is the SMAP observed emissivity (or *T*_*b*_). The resulting SMAP FW retrievals were derived on a daily basis for each 36-km grid cell, consistent with the SMAP L1CTB global EASE-grid format. The inundation area was calculated using the temporal increase of FW extent relative to a preflood period for the 36-km grid cells. For the Idai flood, the averaged surface water conditions during March 11–13, 2019 prior to the cyclone Idai landfall on March 15 were used to describe the preflood inundation level. The increase in FW extent for March 17–19, 2019 relative to the preflood period quantified the newly flooded area due to the cyclone-driven rainfall.

### D. Machine Learning Based Satellite Flood Forecast

**Fig. 2.**
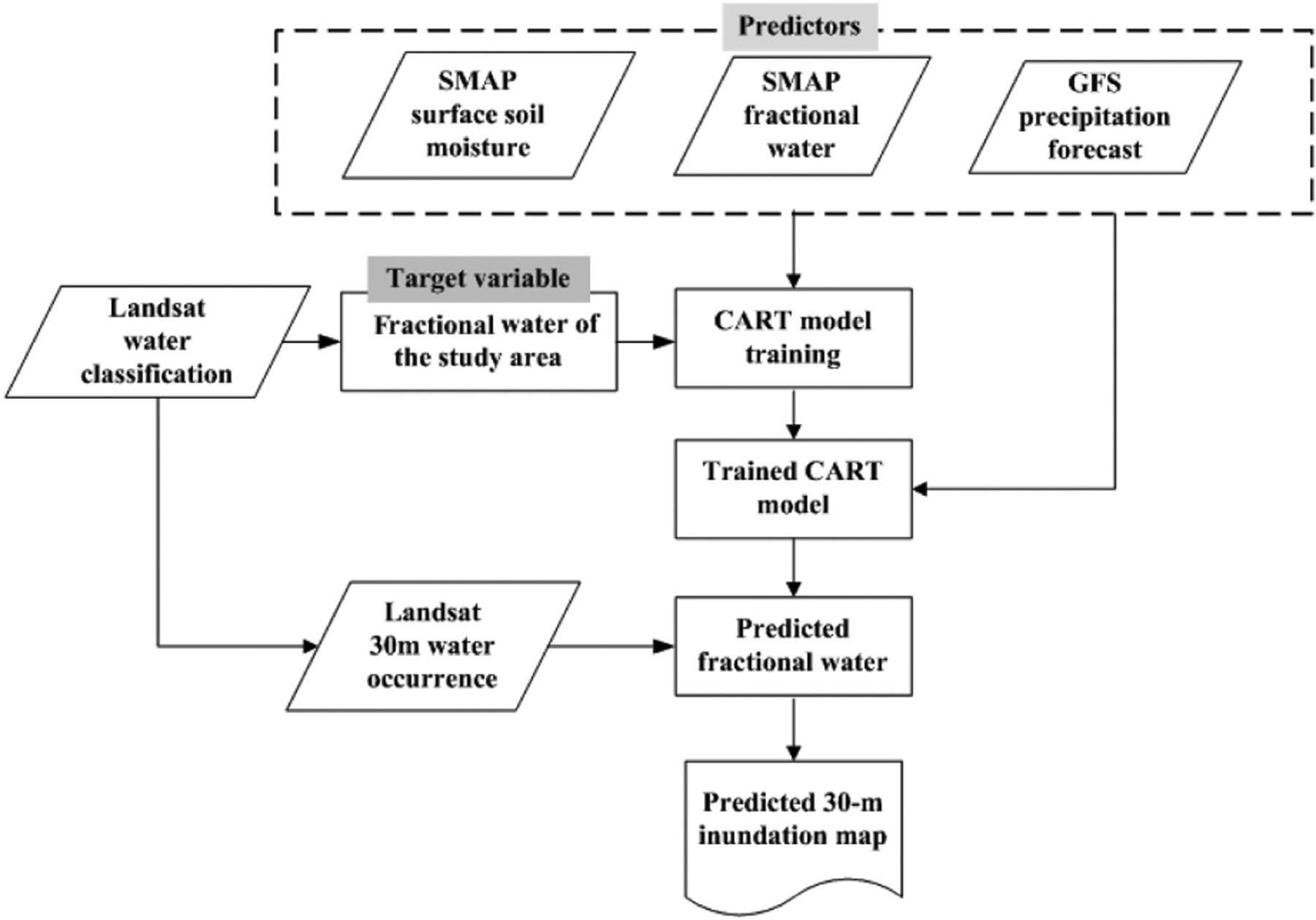
Algorithm flowchart for machine learning based satellite flood forecast and inundation mapping.

Rainfall-driven flood inundation patterns are primarily governed by soil infiltration and saturation excess runoff mechanisms, whereas inundation spatial variability is controlled by topography, soil, rainfall, and vegetation properties [55]. For establishing precipitation and inundation relationships using data-driven approaches, a major assumption of the flood forecast is that precipitation is the primary driver of flooding represented by the satellite observed inundation extent and that these relationships are consistent between model training (past) and forecast (future) periods. Accordingly, historical satellite inundation observations together with model precipitation predictions enable flood inundation forecasts as demonstrated in the algorithm flowchart (Fig. 2) and detailed below.

Our analysis was performed using the GEE platform, which is a web-based service capable of efficient archiving, processing, visualizing, and analyzing petabyte data. The high-performance cloud computation capabilities of GEE enable both conventional spatial analysis and machine learning from a large collection of datasets including remote sensing imagery, reanalysis data, and vector data, and for clarifying their interconnections. Similar to flood predictions based on hydrological models [56], the potential response of surface inundation to projected rainfall depends on initial soil wetness conditions. The SMAP products and previous precipitation information were used to quantify prior surface and soil wetness levels for the study areas and larger domain potentially contributing to the flood inundation. Our data-driven model is region-specific; so only the time-variant features were used as predictors while implicitly accounting for the impacts from static variables such as soil properties and terrain (DEM).

Here we selected the Classification and Regression Trees (CART) model implemented using GEE to derive 1-day (24-h) ahead forecasts of FW inundation patterns within the five Pungwe basin unit catchments [Fig. 1(b)]. The CART model is a decision-tree type machine-learning approach, which is analytically and mathematically rigorous and capable of establishing relationships between target variables and predictors through a recursive partitioning procedure [57], [58]. The CART mechanism allows for automatic missing value handling, cost-sensitive learning, dynamic feature construction, and probability tree estimation [57]. For training and validating the CART model, the GFS, SMAP, and clear-sky Landsat water mask data were collected for the period from May 2015 to February 2019, where 80% of the ~100 data records covering different dates were used for model training and the other 20% for validation. Metrics including correlation coefficient (*R*), root mean square error (RMSE), and RMSE normalized by mean value (nRMSE) were calculated by comparing predicted and observed FW values and used for evaluating model performance. The relative importance of each predictor was determined based on the decrease in node impurity derived during the model training process [57].

Landsat observations acquired at about 10:00 A.M. local time were used in our forecast model, while 8:00 A.M. (UTC time; or 10:00 A.M. local time in Mozambique) was set as the time for predicting catchment FW values. Here, we defined day 0 as the “current” date to make the forecast, and day +*n*/−*n* as the date *n* days after or before day 0. The CART model predictors for the 1-day inundation forecast included the following:

1. cumulative surface precipitation forecasted by the GFS for the 32-h period before 8:00 A.M. (UTC time) of the forecast date or day +1 over the selected catchments and adjacent 50-km buffer zones within the Pungwe River basin (GFS_A32h);
2. cumulative surface precipitation obtained by GFS outputs for the 24-h period of day −1 over the selected catchments and adjacent buffer zones (GFS_B24h);
3. mean SMAP FW over the three-day period before the forecast date over the selected catchments (FW_sc) and buffer zones (FW_bz);
4. NASA-USDA SMAP global surface soil moisture for the study area and buffer zones (SSM_bz).

We excluded the SSM and precipitation forecasts as predictors due to their negligible importance (~0%) in the flood forecasts over the selected catchments. The target variable for the flood forecast is the FW aggregated from the 30-m Landsat water mask for the selected catchments.

We also performed a 3-day (72-h) forecast test to evaluate the model potential for longer term assessments. The associated long-range predictors were defined similar to the 1-day forecast except that cumulative surface precipitation forecasted by GFS for the 80-h period before 8:00 A.M. (UTC time) of day +3 was used (GFS_A80h) instead of GFS_A32h.

The predicted FW values were downscaled for generating 30-m inundation maps using an empirical interpolation approach guided by 30-m water occurrence information derived from the long-term USGS Landsat water mask (Section II-B) [21]. The water occurrence information was used for prioritizing the predicted FW allocation sequentially to all 30-m pixels within the selected catchments. The approach was initially developed for 30-m downscaling of coarse (36-km) grid SMAP FW retrievals, whereby the 30-m results showed favorable spatial accuracy for water (70.71%) and land (98.99%) classifications relative to independent Landsat-8 results over diverse climate, vegetation, and terrain conditions [21]. The resulting 30-m flood inundation forecasts were compared with contemporaneous ARIA SAR derived inundation patterns for independent assessment.

## III. Results

### A. SMAP Flood Mapping

**Fig. 3.**
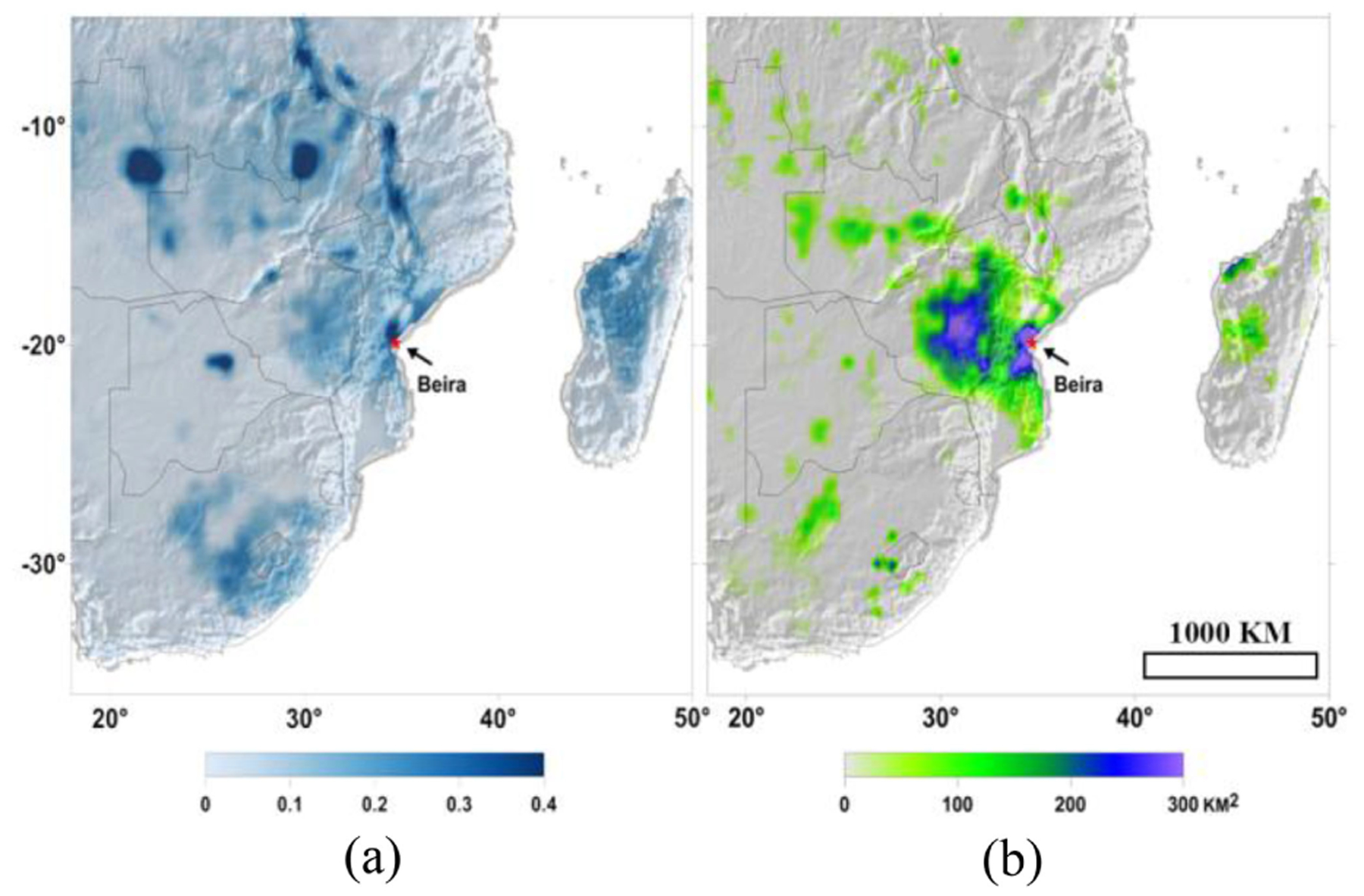
(a) FW extent during peak flood conditions for March 17–19, 2019 depicted by SMAP. (b) Dramatic flooded area increase estimated from SMAP FW retrievals for the 36-km grid cells relative to the period of March 11–13 around the major city of Beira and the surrounding region.

The SMAP L-band microwave radiometer is optimal for flood mapping from cyclone events characterized by heavy cloud cover and intense precipitation. The surface water inundation was depicted by SMAP FW observations for March 17–19, 2019 [Fig. 3(a)], when extensive inundated areas were identified in the southeast African countries including Mozambique, Zimbabwe, Malawi, and Madagascar [59]. Relative to the preflood period, the dramatic flooded area increase [blue and purple shades in Fig. 3(b)] around the major city of Beira and the surrounding areas stemmed from the intense cyclone-driven rainfall event. Severe floods were also detected by SMAP in eastern Zimbabwe where riverine and flash flooding were reported [60]. It is noted that the region was affected by extended rainfall leading up to the cyclone making landfall [61], which likely predisposed the region to flooding. The dark blue areas [Fig. 3(a)] are large lakes (e.g., Lake Bangweulu and Lake Malawi) and seasonal flooded savanna (e.g., Cameia National Park [62]). The newly flooded areas cover about 27 560.6 and 31 400.2 km^2^ for Mozambique and Zimbabwe, respectively, due to rainfall following Idai’s landfall.

### B. Flood Inundation Forecast

**Fig. 4.**
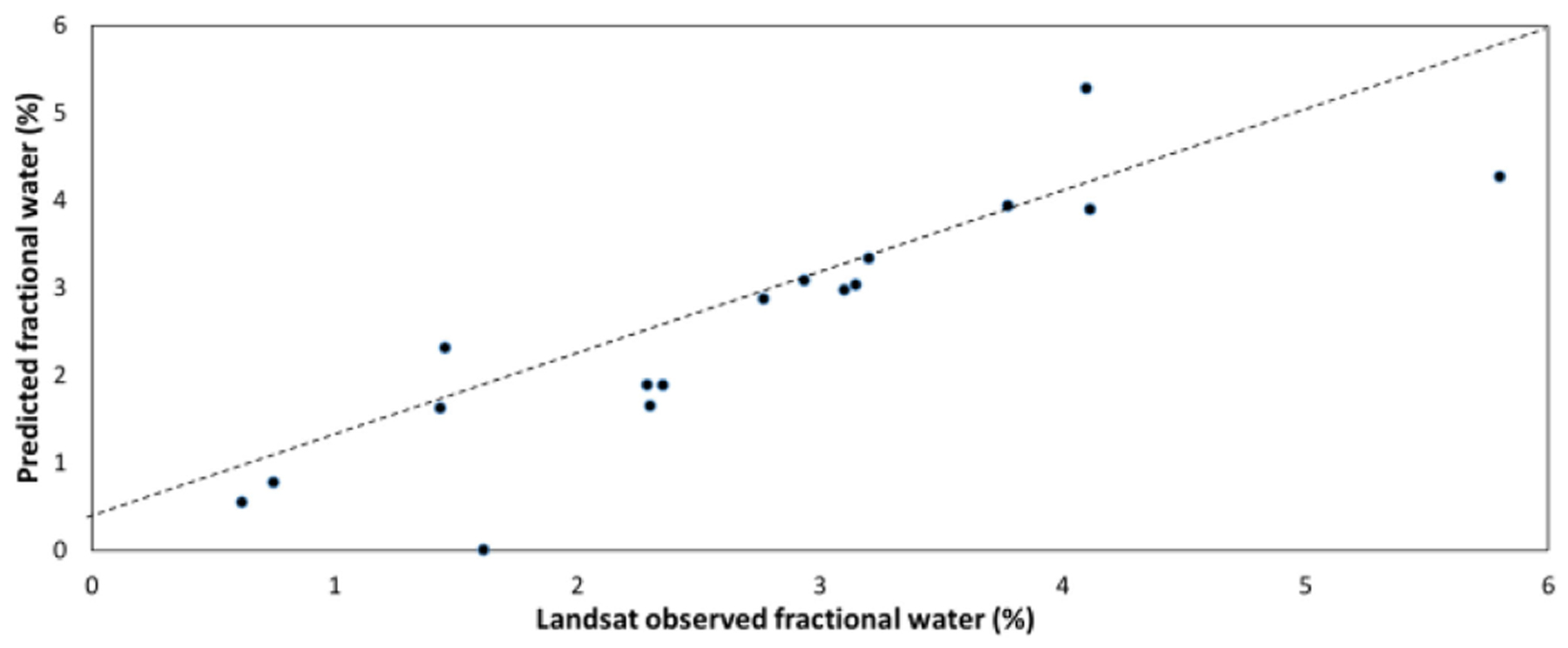
Comparisons between FW data observed by Landsat and predicted by the 1-day forecast CART model for the 163 km^2^ study area within the Pungwe basin using the validation dataset covering randomly selected dates (*R* = 0.87; RMSE = 0.68%, nRMSE = 25.6%).

The 1-day ahead forecast model validation showed predicted FW values consistent with Landsat observations (Fig. 4; *R* = 0.87, RMSE = 0.68%; nRMSE = 25.6%). The relative impact of the model flood forecast predictors, scored from most to least importance were: FW_sc (0.36), SSM_bz (0.34), GFS_B24h (0.18), GFS_A32H (0.06), and FW_bz (0.05). The prior surface water condition over the unit catchments and soil moisture over the larger region had the greatest influence on the 1-day inundation forecast; whereas, the inundation changes after day 0 also depended on precipitation since day −1, along with a relatively small contribution from FW_bz. We then made 1-day flood forecasts using the trained model for the Idai flood peak (March 19, 2019) and recession (March 23, 2019) periods. Accordingly, 76.3% of the unit catchments were predicted as flooded on March 19, 2019, which suggests intensive flood inundation in the region and resembles the Sentinel-1 SAR estimates (82.2%). For March 23, the predicted FW area sharply dropped to 28.8%, which reflects the flood water receding and agrees with the PALSAR result (31.3%).

Compared with the 1-day forecast model, the 3-day forecast validation showed lower correspondence (*R* = 0.53) between predicted and observed FW values, along with larger RMSE 1.39% and nRMSE 58.19% differences. The order of importance of the model predictors was: SSM_bz (0.43), FW_sc (0.19), FW_bz (0.16), GFS_A80h (0.15), and GFS_B24h (0.06). Relative to the 1-day forecast model, FW_sc and GFS_B42h showed less control on the inundation forecast, while accumulated precipitation for the study area after day 0, and surface water and soil wetness over the surrounding region played a more important role in the forecast. We also applied the 3-day prediction model to the Idai event, and the predicted FW values (62.45% for March 19 and 16.85% for March 23) were underestimated by about 24.0% and 46.2% relative to the SAR observations.

**Fig. 5.**
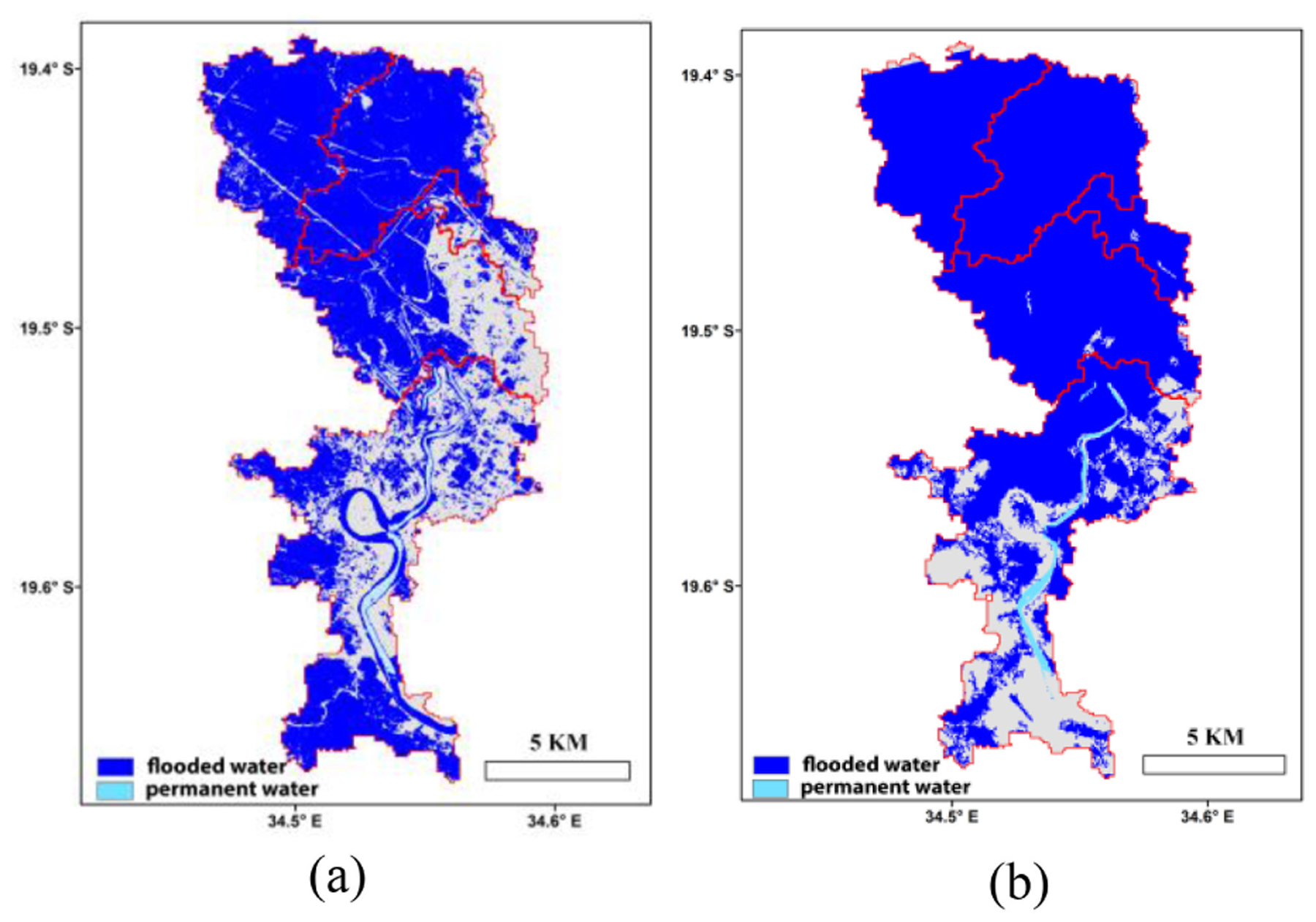
Inundation maps for March 19, 2019, produced using (a) our machine learning based approach and (b) ARIA based on Sentinel-1 SAR observations. Areas without flooding are shown in gray, while red lines denote catchment boundaries.

**Fig. 6.**
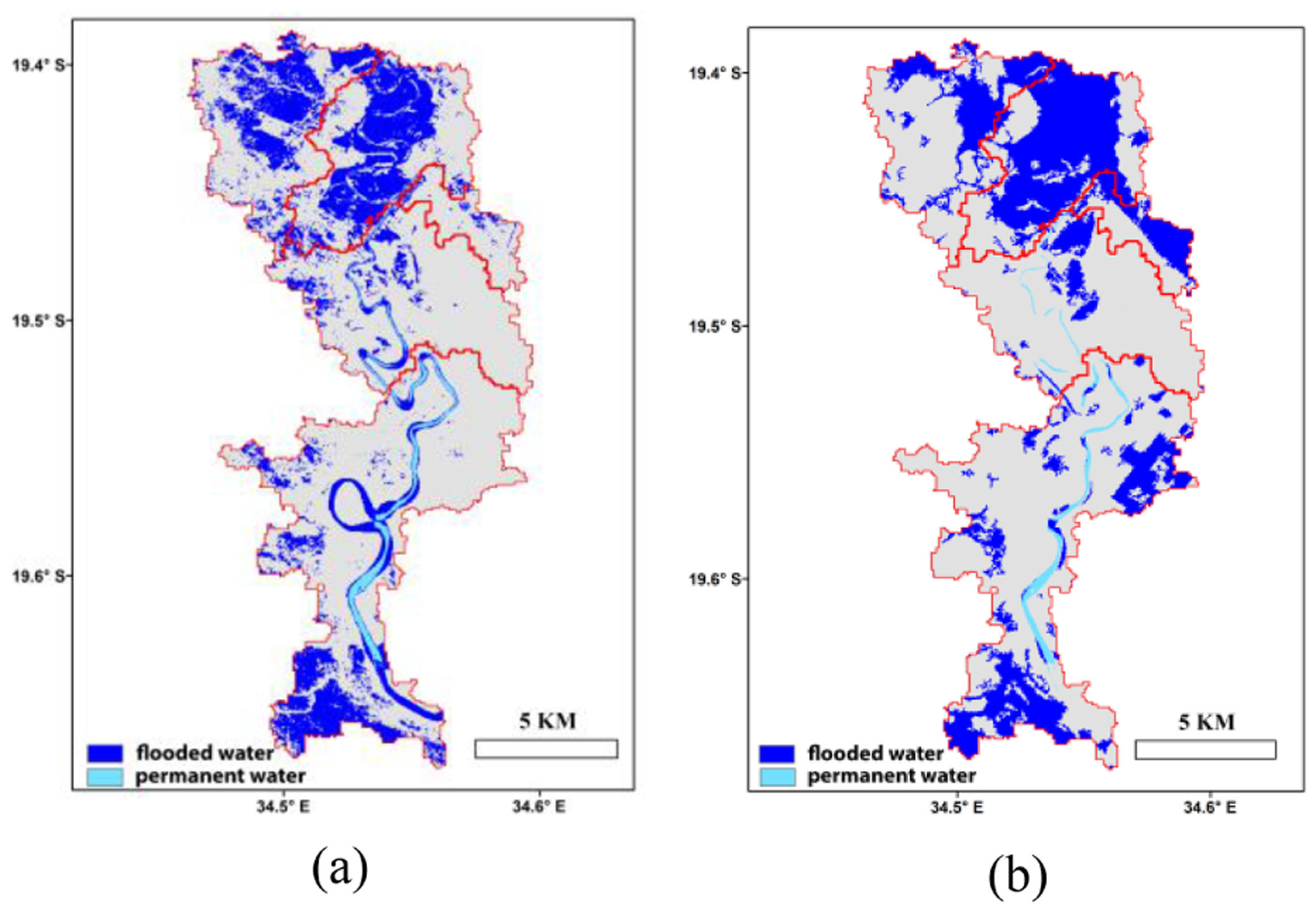
Predicted (a) and observed (b) flood inundation maps for March 23, 2019. The inundation map (b) produced by ARIA was based on ALOS PALSAR observations. Areas without flooding are shown in gray and red lines denote catchment boundaries.

The predicted FW values were further downscaled based on the historical water occurrence map, which indicated higher flood probability in the northern catchments, especially for the area adjacent to the Pungwe river, and lower flood probability in the eastern catchments [Fig. 1(b)]. The 30-m inundation map downscaled from the 1-day forecast for March 19 [Fig. 5(a)] showed the northern catchments as heavily flooded, which was also observed from the ARIA Sentinel assessment. The two line features in the northern part of the basin are major roads in the region, which were not predicted as flooded [Fig. 5(a)]. The associated 30-m inundation map downscaled from the FW forecast for March 23 correctly predicted flooded areas remaining in the northern and southern parts of the study area, consistent with the ARIA PALSAR assessment (Fig. 6). Pixel-based comparisons with the SAR results showed respective commission and omission errors for the 30-m water predictions as 16.5% and 28.8% for March 19, and 43.6% and 49.7% for March 23.

## IV. Discussion

The GEE-based analysis showed the potential of data-driven models in making fine-scale flood inundation forecasts in a data sparse region using complementary global satellite observations and NWPs as key model predictors. The resulting 1-day (24-h) and 3-day (72-h) model forecasts predicted widespread inundation from the Idai cyclone landfall event on March 19 and the subsequent flood recession on March 23. The 3-day model forecast skill was meaningful but lower than the 1-day forecast in terms of correlation and RMSE performance relative to the Landsat reference. This is expected since the GFS predictions have generally lower performance with longer lead time [63]; larger uncertainties likely stem from a lack of satellite surface wetness observations closer to the forecast dates. The SMAP FW and SSM records were the two most important features in the 1-day forecast, which suggests that the background surface wetness level is generally crucial in determining how the coming precipitation affects short-term (e.g., 1 day) inundation changes and potential flood risk. Compared with the 1-day forecast, current soil wetness conditions over the surrounding areas become more important in the 3-day forecast, which suggests the possible contribution from upstream runoff to the downstream flooding. The cumulative precipitation over a longer time period (e.g., the next 80 h) also shows more importance in regulating inundation relative to shorter period precipitation (e.g., 32 h).

The CART model has the advantage of describing complex and nonlinear correspondence between predictors and target variables [64]. However, the regression tree model is built on locally optimal splits, which may lead to relatively less stable predictions over variant training datasets compared with more complex deep-learning methods [64], [65]. One limitation of our study involves the relatively small data sample population (~100) used for training and validating the CART models, which were built from a relatively short period (July 2015 to February 2019) when overlapping satellite and GFS forecast records were available. In addition, tradeoffs were made between sample size and Landsat image quality. Possible solutions for increasing the sample size involve using satellite observations over an extended period and introducing other high-quality water mapping products from satellite SAR sensors. Besides possible misclassifications in Landsat water mask data, such as those resulting from overlying vegetation or mixed-pixel issues, additional uncertainties related to Landsat FW aggregated for the catchments may come from partial data loss due to the remaining cloud cover. It is noted that the machine-learning model was built based on Landsat water/land classifications, and cloud-cover constraints only affected the model training but not the SMAP flood mapping or forecast using the model. A more robust machine-learning model is likely to be built using relatively larger data sample size acquired from longer satellite observations to mitigate possible model overfitting. Here, an additional test was made for demonstrating possible model improvement using training data acquired from a longer study period relative to the approach targeting the Idai event. The NASA SMAP L3 Radiometer Global Daily 36 km EASE-Grid Soil Moisture (Version 7) data were first downloaded for the study region. We then followed the same approach described in Section II-D to build the 24-h forecast model, but 1) using the NASA SMAP product in place of the USDA-NASA SMAP product, which ceased updating in GEE after 2020, and 2) using a relatively larger data sample population (~130) acquired from an extended period from July 1, 2015 to April 30, 2021. Comparisons using the validation dataset showed similar performance to the model described in Section III-B. For the model targeting the Idai event (Section III-B), the correlation coefficients (*R*) between the model predictions and Landsat FW data are 0.94 and 0.87 for the respective training and validation datasets, while the corresponding *R* values for the model updated over the extended period are 0.91 and 0.89. These results indicate more reliable model performance when trained using the larger data sample and longer satellite record. Further model improvements are expected using longer term satellite observations and weather forecast training data, along with more complex machine-learning approaches able to exploit spatial and temporal pattern recognition, such as convolutional neural network (CNN) methods [66]. The potential of machine-learning methods can be further explored by estimating regional inundation directly using multifrequency Tb observations from space-borne microwave radiometers and developing flood inundation forecast models targeting 30-m pixels though such tests have constraints under GEE, which is a noncommercial platform and has a per-user quota on computational resources.

The downscaled flood forecasts provided 30-m inundation mapping consistent with the SAR results. The downscaling analysis for the Idai event benefits from the fact that pixel-based water occurrence information is likely reliably derived from the long-term Landsat record for the region, where frequent floods and droughts have occurred. However, the downscaling approach was constrained by several factors, including SMAP and Landsat surface water detection limitations over dense vegetation, and recent flooding extremes exceeding the historical satellite record [21]. Additional ancillary information including preferential inundation areas and flow networks delineated from digital terrain and surface hydrography data may help improve the downscaling algorithm. In addition, reconstructed water occurrence data with greater weighting to more recent observations may improve downscaling performance. The difference identified in inundation mapping for major roads (Fig. 5) may result from the difficulty of SAR observations in distinguishing water from other low backscattering features such as roads [8]. Part of the inconsistency between the flood forecasts and SAR observations may also result from the different timing of the retrievals in sampling the dynamic surface water conditions. The forecast is made for 10:00 A.M. (local time) when Landsat daytime observations were acquired for CART model training, while the Sentinel-1 and ALOS-2 Idai flood mapping results are derived from, respectively, 18:00 and 12:00 P.M. local time observations.

## V. Conclusion

The SMAP FW data effectively captured surface water dynamics during the severe tropical cyclone Idai event, indicating potential utility for regional flood monitoring to inform disaster assessments. The regional inundation and soil moisture information acquired from SMAP was further combined with Landsat observations and GFS precipitation forecasts to establish a GEE-based machine-learning approach for effective regional flood forecasts. The resulting 1-day (24-h) FW forecast predictions were highly correlated (*R* = 0.87) with contemporaneous Landsat observations and showed relatively low errors (RMSE = 0.68%; nRMSE = 25.6%). A model feature importance analysis showed that timely satellite measurements of surface wetness over the study area are crucial for determining the 1-day forecast inundation extent from a rainfall-driven flood event, while the cumulative precipitation over a longer period and surface wetness information for the surrounding region become more important for longer (3-day) forecasts. The 1-day forecasts for the Idai event captured the flood inundation temporal dynamics and 30-m spatial pattern consistent with independent satellite SAR observations. The approach provides new capacity for global flood monitoring and forecasts from synergistic satellite observations, including data sparse regions of Africa.
